# Influence of OCT2 gene variants on metformin efficacy in type 2 diabetes: insights into pharmacogenomics and drug interactions

**DOI:** 10.1186/s12967-025-06720-y

**Published:** 2025-08-07

**Authors:** Ma’mon M. Hatmal, Omar Abuyaman, Mohammad A. I. Al-Hatamleh, Heba Tayyem, Amin N. Olaimat, Ali Mussa, Iman Aolymat, Aymen Abuawad, Mohanad Odeh, Rana Qawaqzeh

**Affiliations:** 1 Department of Medical Laboratory Sciences, Faculty of Applied Medical Sciences, The Hashemite University, P.O. Box 330127, Zarqa, 13133 Jordan; 2 Department of Pathology, School of Medicine, Stanford University, 300 Pasteur Drive, Stanford, CA 94305 USA; 3 UPMC Hillman Cancer Center, Division of Malignant Hematology and Medical Oncology, Department of Medicine, University of Pittsburgh School of Medicine, Pittsburgh, PA USA; 4 Department of Clinical Nutrition and Dietetics, Faculty of Applied Medical Sciences, The Hashemite University, Zarqa, Jordan; 5 Department of Hematology, School of Medical Sciences, Universiti Sains Malaysia, Kubang Kerian, Kelantan, Malaysia; 6 Department of Biology, Faculty of Education, Omdurman Islamic University, Omdurman, Sudan; 7 Department of Anatomy, Physiology and Biochemistry, Faculty of Medicine, The Hashemite University, Zarqa, Jordan; 8 GenoLabs, Amman, Jordan; 9 Department of Clinical Pharmacy and Pharmacy Practice, Faculty of Pharmaceutical Sciences, The Hashemite University, Zarqa, Jordan; 10 Faculty of Medicine, The Hashemite University, Zarqa, Jordan

**Keywords:** T2DM, Insulin, Organic cation transporters, SNP, *SLC22A2*, 808G > T, Rs316019

## Abstract

Metformin, a widely prescribed treatment for type 2 diabetes mellitus (T2DM), demonstrates significant inter-individual variability in its therapeutic response. This variability is potentially driven by genetic differences in drug transporters. Among these transporters, the organic cation transporter 2 (OCT2) plays a critical role in the pharmacokinetics of metformin by mediating its uptake into renal epithelial cells for excretion. This review explores the potential impact of genetic variations in OCT2 gene (*SLC22A2*) on the pharmacokinetics and pharmacodynamics of metformin. These genetic variations can alter metformin accumulation in the kidneys, impacting its overall clearance and therapeutic effectiveness. Furthermore, the interactions of metformin with other drugs, especially in T2DM patients, can compromise its pharmacokinetics. Thus, it is important to consider the influence of genetic variability and potential drug interactions when prescribing metformin. Incorporating genetic testing into clinical decision-making could help optimize dosing strategies and improve treatment outcomes, particularly when managing patients with complex comorbid conditions.

## Introduction

Chronic hyperglycemia is caused by abnormalities in the insulin secretion, activity or a combination of both in a group of metabolic illnesses collectively referred to as diabetes mellitus (DM) [5, 124]. According to estimates from the International Diabetes Federation (IDF), approximately 536.6 million adults worldwide (10.5% of the population) were living with diabetes in 2021. The IDF projects that this number will increase to 783.2 million adults (12.2% of the population) by 2045 [112]. The dysfunction and loss of insulin-secreting pancreatic beta cells are closely linked to the development of DM [7, 14, 47, 130]. DM is primarily classified into two main types: type 1 and type 2. Over 90% of global diabetes cases are attributed to type 2 DM (T2DM), which results from a combination of environmental and genetic factors [51, 56, 71, 88, 119, 121].

Single nucleotide polymorphisms (SNPs) are genetic variations that can influence cellular processes, modify drug responses, impact disease susceptibility and affect sensitivity to environmental factors [50]. Pharmacogenomics research on anti-diabetic medications, including metformin, has shown that gene polymorphisms are closely linked to different responses to these treatments [27, 91, 137]. For example, polymorphisms in the organic cation transporter 2 (OCT2) gene play a critical role in the therapeutic use of metformin by altering gene function, which in turn enhances the transport of cationic compounds, including anti-diabetic medications.

This work provides a comprehensive overview of the role of OCT2 and its genetic variations in the treatment of T2DM with metformin. The review delves into how these genetic differences can influence absorption, distribution and elimination of metformin, ultimately impacting treatment efficacy and patient outcomes. Additionally, we discuss the potential drug interactions and the importance of personalized medicine in optimizing metformin therapy. By enhancing the understanding of the role of OCT2 in metformin pharmacogenomics, this work paves the way for more research on targeted and effective therapeutic strategies in the management of T2DM.

### T2DM

The World Health Organization (WHO) estimates that approximately 422 million people worldwide are living with diabetes, with the majority residing in low- and middle-income countries. Importantly, diabetes is directly responsible for up to 1.5 million deaths annually [29]. In the past few decades, there has been a consistent rise in both the incidence and prevalence of diabetes [29]. Additionally, geographical location appears to have an impact on the prevalence and frequency of T2DM [37].

**Fig. 1:**
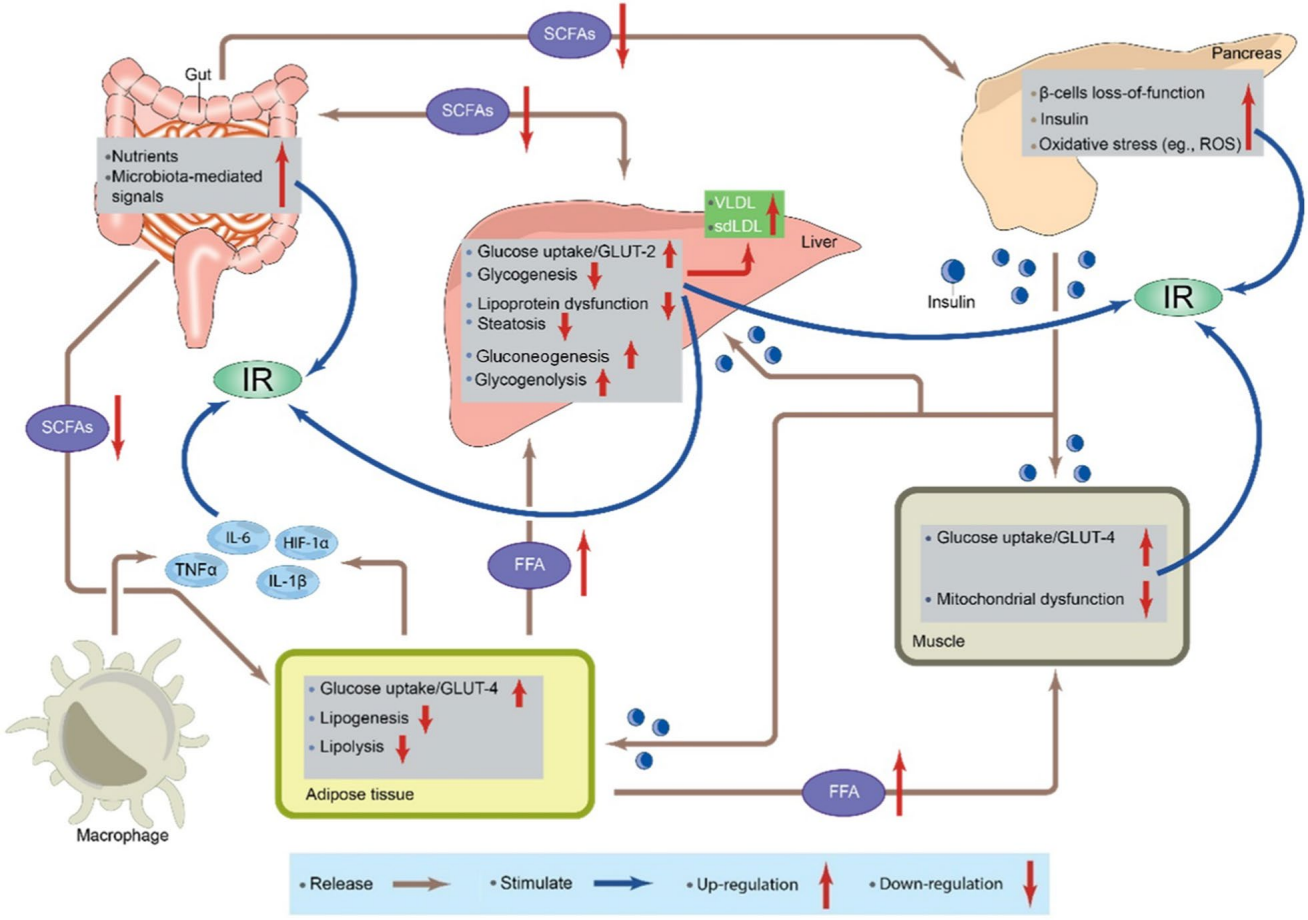
Diagrammatic representation of T2DM pathophysiology. The schematic highlights the interplay between key organs and the molecular mechanisms driving insulin resistance (IR). SCFAs, short-chain fatty acids; VLDL, very low-density lipoproteins; sdLDL, small dense low-density lipoproteins; FFA, free fatty acids; GLUT-2, glucose transporter type 2; GLUT-4, glucose transporter type 4; IL-6, interleukin 6; HIF-1α, hypoxia-inducible factor 1-alpha; TNFα, tumor necrosis factor alpha; IL-1β, interleukin 1 beta

The two main causes of T2DM are decreased insulin synthesis by pancreatic beta cells and reduced tissue insulin sensitivity. Dysregulated and dysfunctional insulin secretion results in impaired insulin production, which disrupts glucose homeostasis [104]. In insulin resistance, the ability of muscles and adipose tissue to uptake and utilize glucose is reduced, while glucose production in hepatocytes is increased. This process is regulated by insulin, which prevents glycogenolysis in the liver [29, 37, 86, 93]. In the absence of the insulin-glycogenolysis feedback mechanism, the liver continues to produce glucose, leading to hyperglycemia and the development of T2DM [100]. The pathophysiology of T2DM is illustrated in Fig. 1.

Numerous genetic variants have been consistently identified in association with T2DM, highlighting the complex genetic underpinnings of the disease. In addition, other variants have been associated with diabetes-related traits, such as insulin resistance, beta-cell dysfunction and impaired glucose metabolism [31, 96]. These genetic factors contribute to the variability in disease onset, progression, and response to treatment among individuals. Therefore, continued exploration of these genetic variants holds the potential for improved risk prediction, early diagnosis and personalized therapeutic strategies for T2DM [62, 111].

For instance, several genetic variations in the calpain-10, the first gene identified in relation to diabetes, and adiponectin genes have been associated with T2DM by influencing insulin function and the amount of glucose produced by the liver [84, 116]. Similarly, multiple studies in different ethnicities have showed that variations in the hepatocyte nuclear factor 4 alpha (HNF4A) gene can reduce the amount of insulin the pancreas produces, increasing the propensity to developing T2DM [11, 71]. The glucagon receptor (GCGR) gene is also reported as a diabetes susceptibility gene because glucagon is an essential hormone for regulating blood glucose levels [51, 88]. Moreover, the effectiveness of T2DM drugs, including metformin, sulfonylureas, dipeptidyl peptidase 4 (DPP-4) and sodium-glucose transport protein 2 (SGLT-2) inhibitors, may vary between individuals due to several factors including heredity, age, gender, hypertension and others [86]. Importantly, studies showed that genetic variables, especially in drug transporter genes, can cause significant interindividual variation in response to metformin, the most widely prescribed drug for treating T2DM [85, 91].

### Metformin

*Galega officinalis*, a herbaceous plant derived from the French lilac, serves as the natural source of metformin, which has emerged as a vital agent in the fight against T2DM [104]. Metformin, which is the only medication in the biguanide class that is readily available, is utilized alone or in combination with other oral hypoglycemic medications [100, 104, 125]. Metformin is available in two formulations: immediate-release (IR) and extended-release (XR). The XR formulation offers a longer half-life and lower peak drug concentrations, enabling once-daily dosing. This improved dosing regimen enhances patient compliance and overall treatment experience compared to the multiple daily doses required with the IR formulation [113].

**Fig. 2:**
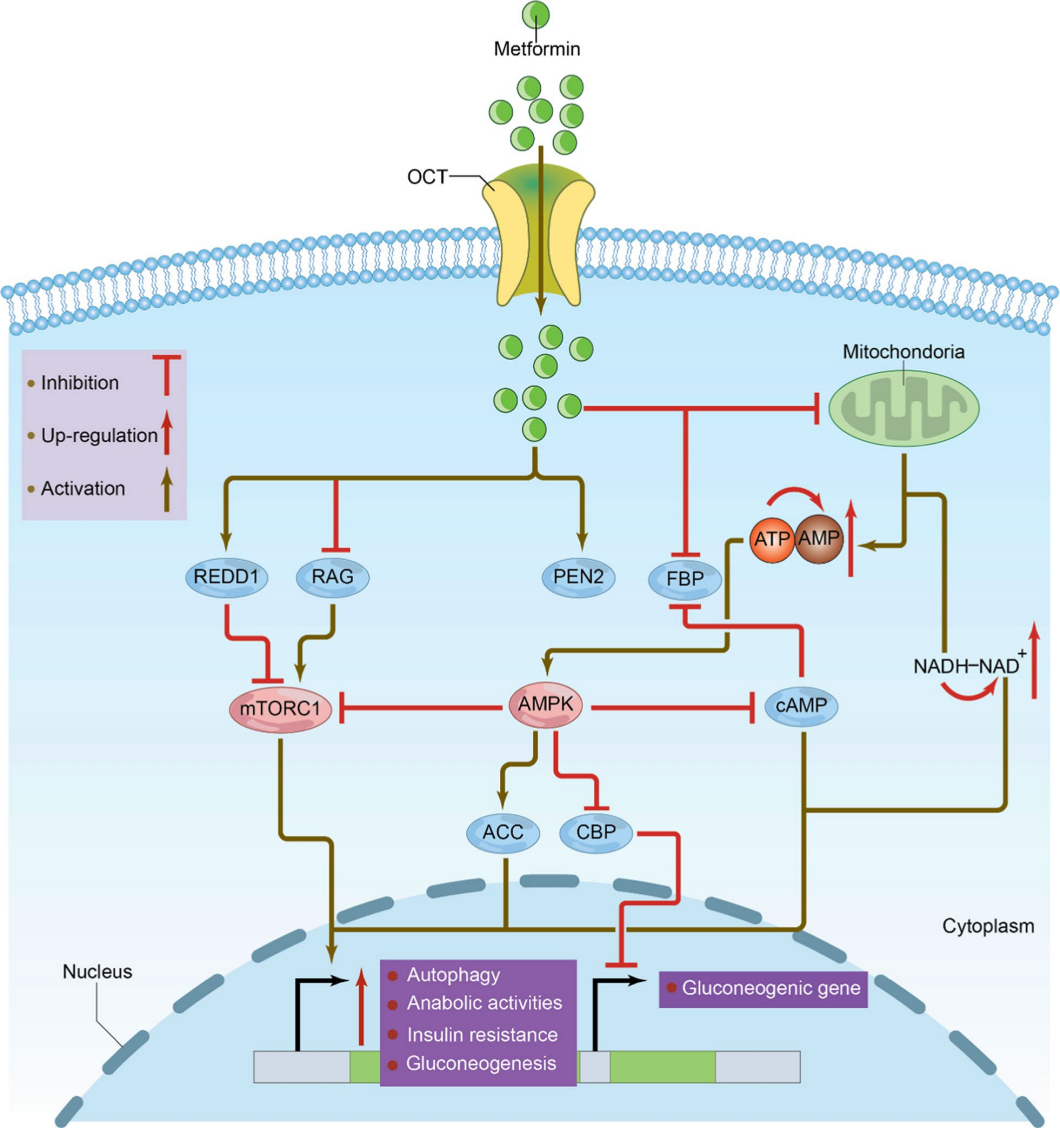
Mechanistic representation of the pathways influenced by metformin in regulating glucose levels. Metformin is primarily transported into cells by organic cation transporters (OCTs). It activates adenosine monophosphate (AMP)-activated protein kinase (AMPK) through lysosomal signaling or mitochondrial energy modulation, leading to several downstream effects that enhance insulin sensitivity and reduce blood glucose levels. Activation of AMPK inhibits acetyl-CoA carboxylase (ACC), which reduces lipid synthesis and promotes fatty acid oxidation. It also suppresses gluconeogenic gene expression via CREB-binding protein (CBP), thereby reducing hepatic glucose production. Furthermore, AMPK activation inhibits the mammalian target of rapamycin complex 1 (mTORC1), curbing anabolic processes such as protein and lipid synthesis, and promotes autophagy, which improves cellular energy balance. Additionally, metformin decreases gluconeogenesis by reducing cAMP levels, which plays a critical role in the regulation of glucose production. While AMPK activation represents a central mechanism of metformin action, it is not the sole pathway involved. Metformin also exerts AMPK-independent effects, including inhibition of mitochondrial complex I, modulation of cellular energy status via changes in NADH/NAD⁺ and ATP/cAMP ratios, and direct suppression of fructose-1,6-bisphosphatase activity. These complementary mechanisms contribute to metformin’s glucose-lowering effects and underscore its pharmacological complexity. Independently of AMPK activation, metformin modulates cellular energy status by altering the cyclic cAMP:adenosine triphosphate (ATP) and nicotinamide adenine dinucleotide hydrogen (NADH):NAD^+^ ratios, thereby inhibiting energy-intensive gluconeogenic pathways. It can also directly target and inhibit fructose-1,6-bisphosphatase (FBP) activity, further contributing to its glucose-lowering effects. REDD1, regulated in development and DNA damage response 1; RAG, recombination-activating gene; PEN2, presenilin enhancer protein 2

Although the precise mechanism of action of metformin remains incompletely understood, it is known that metformin does not stimulate insulin secretion [63]. Instead, it reduces hepatic gluconeogenesis by activating adenosine monophosphate (AMP)-activated protein kinase (AMPK), inhibits the metabolism and transport of specific substrates such as lactate and decreases dietary glucose absorption [1, 34]. These combined effects contribute to improved glucose homeostasis and reduced blood glucose levels in patients with T2DM. Additionally, it enhances the activity of insulin-mediated insulin receptor tyrosine kinase, which facilitates the translocation of insulin-sensitive glucose transporters to the cytoplasmic membrane. This process allows cells to respond more effectively to insulin, thereby improving glucose uptake and significantly boosting insulin sensitivity [49, 60]. Figure 2 illustrates the mechanisms by which metformin regulates glucose levels.

As a hydrophilic chemical with a pKa of 12.4, the free diffusion of metformin through cell membranes is not anticipated. Over 97% of metformin is removed in their undenatured form through urine [118]. The renal clearance of metformin ranges from 443 ml/min to approximately 616 ml/min, which indicates a significant tubular secretion. Patients with renal failure are more susceptible for developing metformin-induced lactic acidosis due to variations in the drug transporters in their kidneys [15, 21, 57, 79, 117, 136].

### Organic cation transporters

**Fig. 3:**
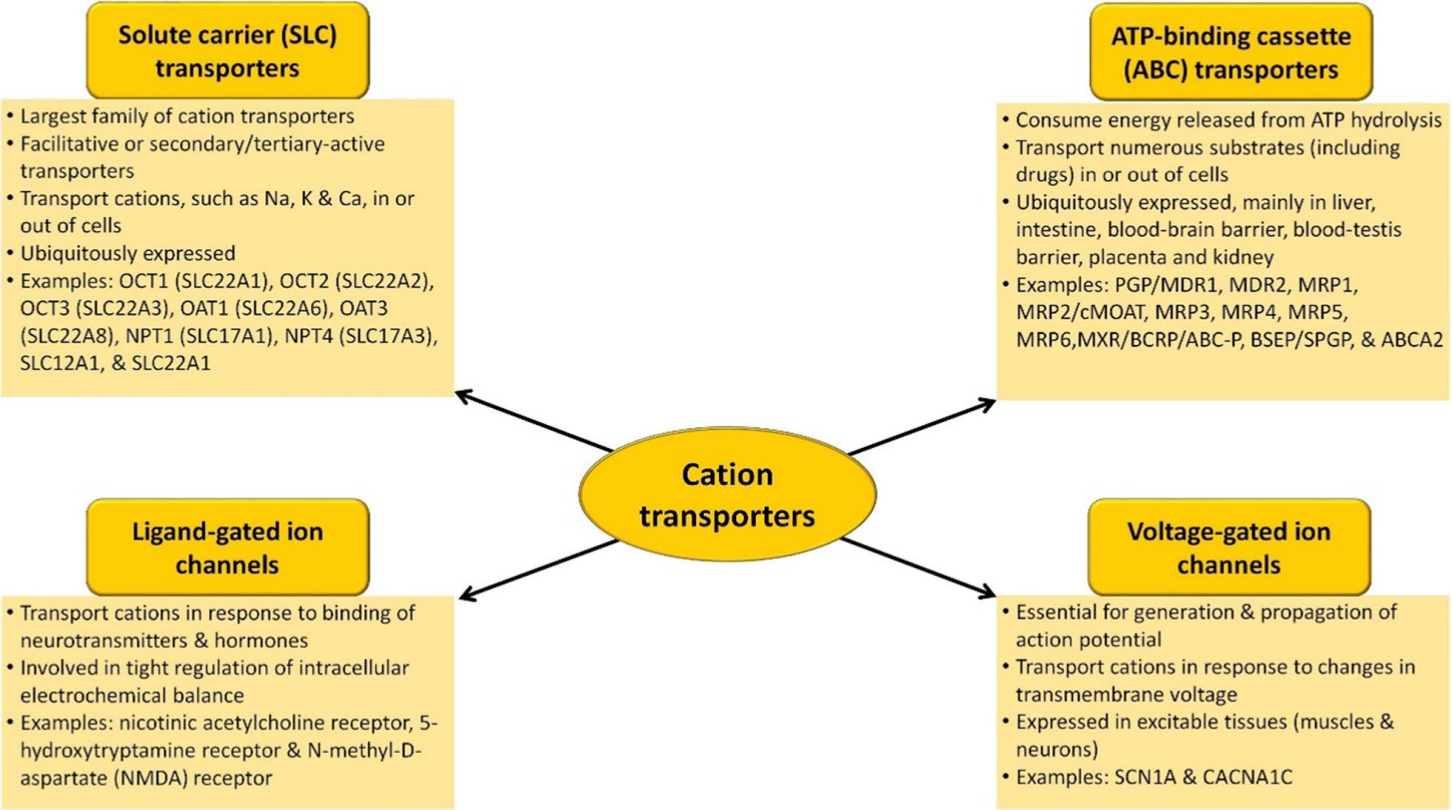
Overview of key characteristics of cation transporter superfamilies

Cation transporters are involved in the movement of positively-charged ions across biological membranes and broadly categorized into several families based on their structural features, transport mechanisms and specific functions [13, 16, 40, 42, 48, 70, 98, 105, 120] (Fig. 3).

As a distinct subgroup within the broader solute carrier (SLC) superfamily, the OCT family consists of three transporter isoforms including OCT1, OCT2 and OCT3 [24]. Interestingly, these transporters run on an electric charge (cations and anions) rather than sodium or proton gradients [101]. The expression of OCTs have been detected in various tissues, including kidney, colon, brain, lung, liver and placenta [59, 82]. OCT1 is primarily expressed in the liver, where it plays a critical role in the hepatic uptake of cationic drugs, influencing their pharmacokinetics and therapeutic effects [66, 81]. OCT2 is the most prevalent isoform in the basolateral membranes of human renal tissue, where it plays a key role in drug and metabolite transport [26, 82]. In contrast, OCT3 is widely distributed across various tissues and is particularly abundant in the mammalian brain, where it contributes to neurotransmitter regulation and other physiological processes [39, 129].

**Fig. 4:**
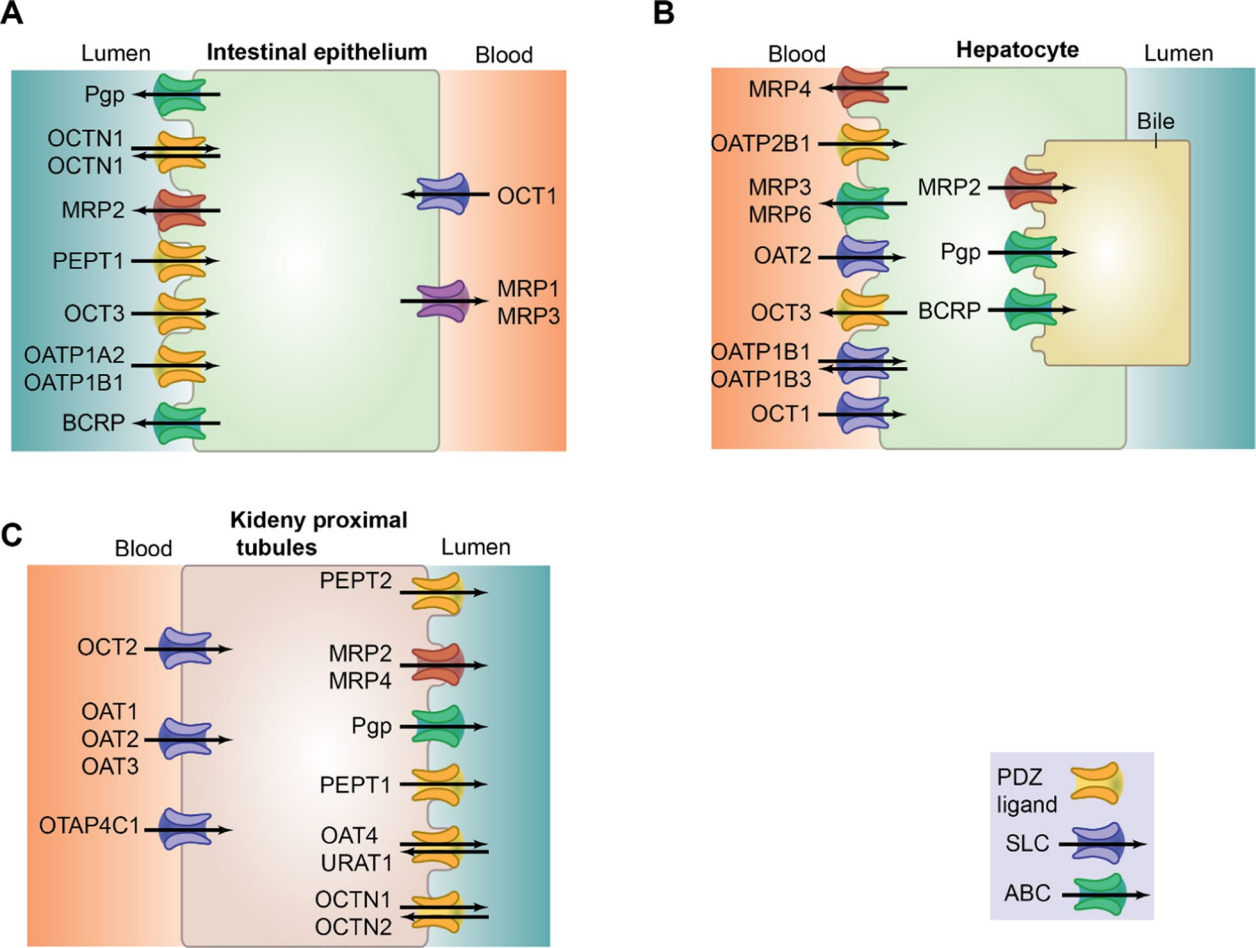
Key drug transporters in the human body. The diagram illustrates transporter localization in **a** intestinal epithelium, **b** hepatocytes and **c** kidney proximal tubules. ABC, ATP-binding cassette; SLC, solute carrier; OCTN, organic cation transporter novel; OCT, organic cation transporter; OATP, organic anion-transporting polypeptide; PEPT, peptide transporter; MRP, multidrug resistance-associated protein; Pgp, P-glycoprotein; BCRP, breast cancer resistance protein; OAT, organic anion transporter; URAT1, urate transporter 1; OTAP4C1, organic anion-transporting polypeptide 4C1

Numerous renal transporters (e.g., OAT1, OAT3, OATP4C1, OCT2, MDR1, MATE1, OAT4, MRP2, and MRP4) have been identified through years of research in humans, monkeys, dogs and rats [144]. The expression of these transporters in the renal tissues vary between different species, sex-genders, ages and disease statuses, suggesting different pharmacokinetics of drugs [52, 74, 144]. Figure 4 summarizes the key human transport proteins involved in drug absorption, distribution, metabolism and excretion across the intestinal epithelium, hepatocytes and kidney proximal tubules. In the human kidney, OAT1, OCT2 and MATE1 are the most abundant transporters, surpassing other drug transporters commonly reported in animals [8].

### Metformin and OCTs

**Fig. 5:**
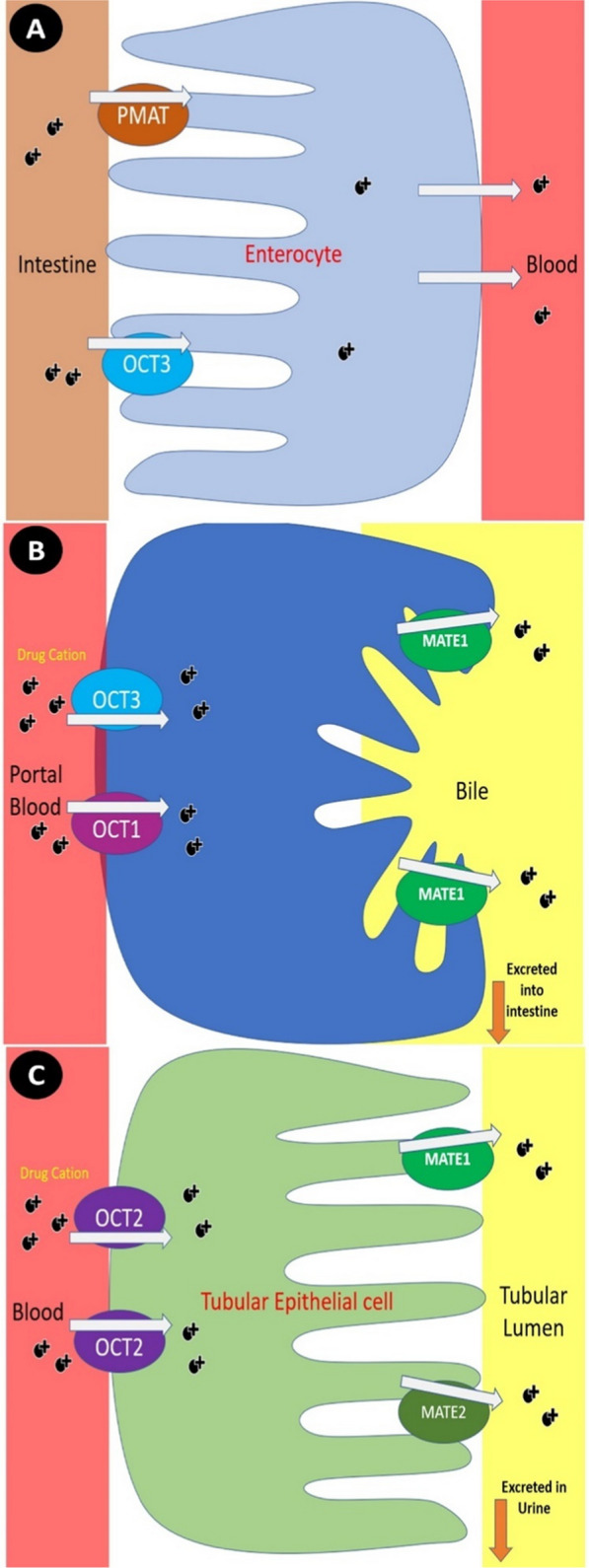
Functions of OCTs and MATEs in the human kidney. OCTs and MATEs are the primary transporters responsible for secreting cationic substances, including drugs, into the urine. **a** Plasma membrane monoamine transporter (PMAT) and OCT3 mediate the entry of drug cations from the intestine into erythrocytes and the bloodstream. **b** OCT1 and OCT3 facilitate the entry of drug cations into the liver, from where they are transported to the intestine. **c** After OCT2 absorbs drugs from the blood at the basolateral membrane of tubular epithelial cells in the kidney, they are released from cells via MATE1 and MATE2-K

OCTs and multidrug and toxin extrusion proteins (MATEs) work together to facilitate the uptake and excretion of drugs, toxins and endogenous metabolites, playing a crucial role in drug clearance, particularly in the liver and kidneys [82]. While OCTs mediate the uptake of organic cations into cells, MATEs act as efflux transporters, expelling these substrates into bile or urine using a proton gradient [61]. Figure 5 illustrates the roles of OCTs and MATEs in drug transport within the human kidney. This sequential interaction ensures efficient elimination of shared substrates, including drugs like metformin. Therefore, dysregulation in this system, whether due to genetic polymorphisms, drug-drug interactions or transporter inhibition, can lead to substrate accumulation, reduced clearance and toxicity.

On the other hand, one of the most intriguing aspects of metformin is the role of genes linked to its therapeutic responses. These genetic factors can significantly influence how individuals metabolize and respond to the drug [86, 91]. Additionally, drug transporters play a crucial role in the pharmacokinetics of metformin, as they govern its absorption, distribution and elimination. These transporters significantly influence its bioavailability and therapeutic efficacy [69, 108]. Hence, investigating genetic polymorphisms in drug transporter genes is critical for understanding the variability in patient responses to metformin. Numerous genes have been linked to the therapeutic efficacy of metformin, including those involved in drug transport, metabolism, and cellular signaling. Key genes include *SLC22A1, SLC22A2, SLC47A1, SLC47A2, SLC22A3, PRPF31, CPA6, PRKAG2, STK11, TCFL2, PPARGC1A, ABCC8, KCNJ11, CAPN10, SP1* and *FMO5* [2, 4, 6, 17, 18, 30, 41, 86, 102, 132]. While achieving personalized and effective long-term management of T2DM remains a significant challenge despite the availability of numerous treatment options, understanding the roles of these genes offers valuable insights into the pharmacogenomics of metformin, potentially overcome this challenge.

OCT1, OCT2 and OCT3 are encoded by the *SLC* genes *SLC22A1*, *SLC22A2* and *SLC22A3*, respectively. These genes share a conserved structure, comprising 11 exons, 10 introns, and 12 transmembrane helices, and are located on chromosome 6q26-q27 [72, 73]. OCT genes are among the most studied transporter genes due to their crucial role in the absorption, distribution and elimination of metformin in the body [18, 92, 107].

Numerous studies have examined the relationship between pharmacokinetics and clinical effects of metformin and genetic variations in OCT genes. The abnormal metformin disposition in patients has been linked to specific SNPs in transporters. Researchers examined the influence of OCT1, OCT2, MATE1 and MATE2 promoter variations on metformin sensitivity, and they found that *SLC47A1* rs2289669 and *SLC47A2* rs12943590 variants could predict resistance to insulin in T2DM subjects under metformin therapy [18]. Furthermore, fasting insulin in T2DM patients taking metformin was significantly impacted by the interaction between dose of metformin (30g) and *SLC47A2* rs12943590. These findings suggest that metformin transport and response among T2DM patients are influenced considerably by promoter variants in *SLC47A1* and *SLC47A2* [18].

### OCT2

**Fig. 6:**
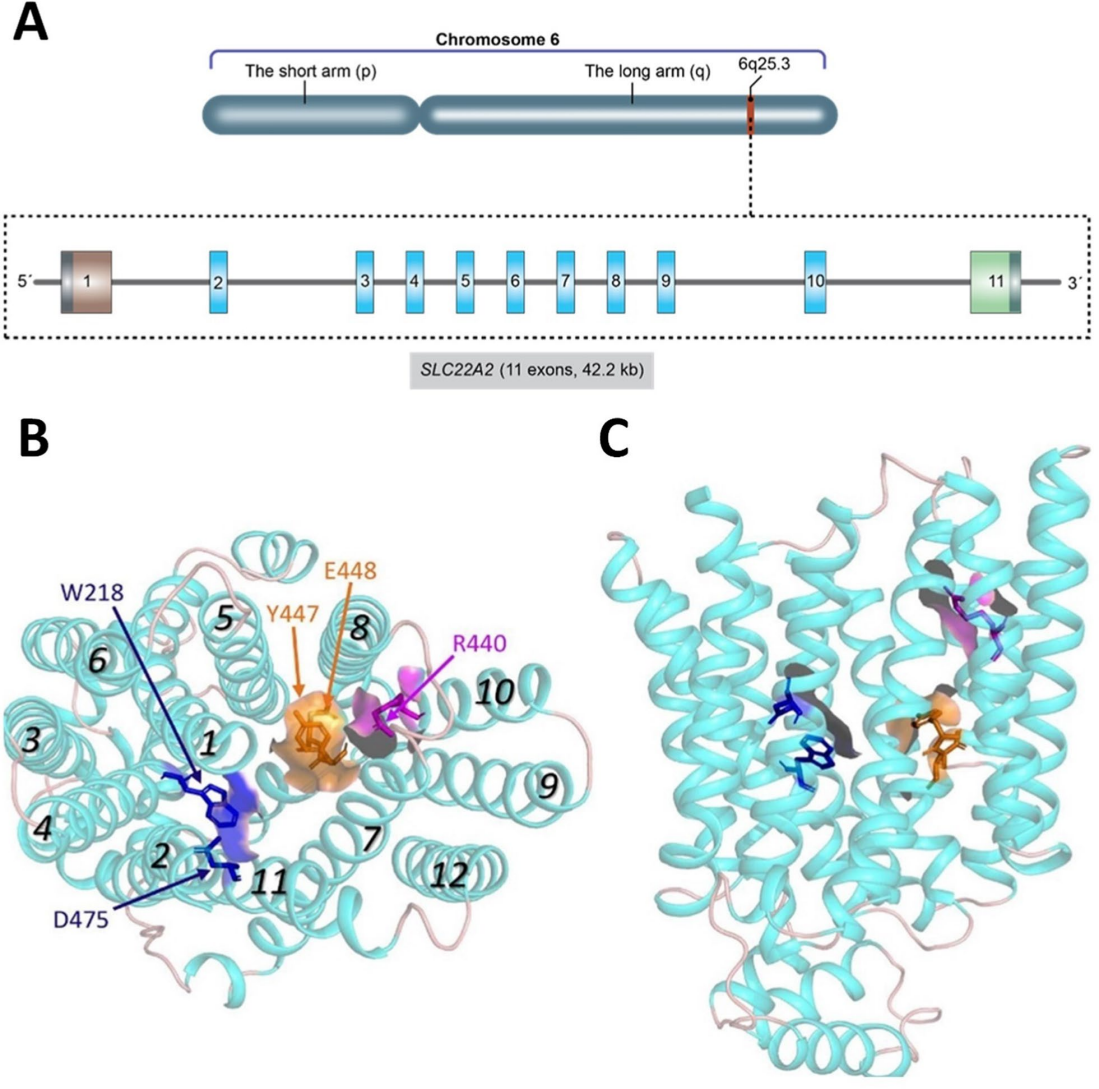
Structural details of the OCT2 gene (*SLC22A2*) and protein. **a** Chromosomal location of the *SLC22A2* gene, with a detailed depiction of the *SLC22A2* gene structure, highlighting its exons and introns. Data was obtained from the human reference genome assembly GRCh38.p14 (GCF_000001405.40), as recorded in the NCBI database. **b** Front view of the extracellular side, and **c** top view of the extracellular component from the side. The transmembrane helices 1–12 are labeled in italics. The unoccluded GLUT3 structure, facing outward, served as the template for the human OCT2 homology model. The 3D structures were adapted from [128]

OCT2 protein, which is encoded by the *SLC22A2* gene and has 555 sequences of amino acids and a mass of 66 kilodaltons (Fig. 6), is active in the basolateral membrane of renal proximal tubule cells. It facilitates the uptake and secretion of tiny, water-soluble molecules with a positive charge under normal pH conditions [118]. Such molecules include various organic cationic drugs, such as those utilized in DM, for instance, metformin, phenformin, sulfonylureas and repaglinide. The electrochemical gradient across the cell membrane controls the direction in which these molecules are transported [118].

It has been reported that elimination of metformin is primarily mediated by OCT2. OCT2 facilitates the uptake of metformin into renal tubular cells, where it is subsequently transported into urine for elimination [57, 58, 76, 95]. However, multiple factors, including age, species and gender, can significantly impact drug pharmacokinetics and influence dosing and therapeutic outcomes. For example, two age groups of rats (3 months and 12 months) were used to study metformin excretion. The two groups exhibited significant differences in t1/2, urine accumulation and total clearance values. This discrepancy is likely due to a substantial age-related decline in OCT2, a transporter essential for the ability of kidney to excrete metformin [99].

Another study demonstrated species-specific variations in OCT2 expression following cisplatin treatment, showing a decrease in OCT2 mRNA and protein levels in mice, while rats exhibited an increase [3]. This suggests that drug transporters undergo dynamic changes throughout the lifespan of an organism, highlighting the importance of this information for the development of new drugs and their therapeutic applications. The OCT2 expression levels in kidneys could be gender-specific, as shown by the fact that male rats and mice have greater levels of OCT2 protein and mRNA in the basolateral membrane of the proximal tubules compared to females [33, 118]. Moreover, OCT2 is also expressed in pancreatic beta cells and may function in the transport of anti-diabetic drugs like metformin [106]. Therefore, it is possible that the genetic variations in the *SLC22A2* gene can alter the function of OCT2, potentially leading to significant changes the pharmacokinetics of metformin.

### Genetic variations in SLC22A2 gene

Since they can alter the pharmacokinetic profiles of several medicines, *SLC22A2* gene variations are potential indicators for precision medicine [68]. The SLC22A2 gene has been found to contain approximately 500 genetic variants, 13 of which result in non-synonymous amino acid changes, with most occurring at a frequency of less than 1% [141]. Table 1 lists the most important *SLC22A2* SNPs.

**Table 1:** Nomenclature and location of common *SLC22A2* SNPs

SNP	Other names	Location on chromosome 6
rs316019	808G > T (A270S)	160,249,250 (exon 5)
rs8177504	160 C > A (P54S)	160,258,598 (exon 1)
rs8177509	481 T > C (F161L)	160,256,651 (exon 2)
rs8177508	493 A > G (M165V)	160,256,639 (exon 2)
rs8177516	1198 C > T (R400C)	160,243,653 (exon 7)
rs8177517	1294 A > C (K432Q)	160,242,388 (exon 8)
rs201919874	596 C > T (T199I)	160,250,625
rs145450955	602 C > T (T201M)	160,250,619
rs138765638	Promotor variant	160,250,625
rs59695691	Promotor variant	160,259,707–160259713
rs10755577	Intron variant 1602-1964G > A	160,219,462
rs17588242	Intron variant 1388 + 96 A > G	160,242,198
rs17589858	Intergenic variant	160,278,084
rs2928035	Intron variant 1602-2351C > G	160,219,849
rs312024	Intron variant 91 + 14158G > C	68,326,963
rs312025	Intron variant 91 + 11361G > A	68,324,166
rs312026	Intron variant 120 + 15421 C > G	21,236,715
rs3127573	5 prime UTR variant −970T > C	160,260,361
rs533452	Intron variant 1297 + 723 T > G	160,276,730
rs662301	Non-coding transcript exon variant 224 C > T	160,275,887
rs2289669	Intron variant 922-158G > A	19,560,030
rs3119309	Intron variant −1296-3353G > A	160,264,040
rs7757336	Intron variant −1296-7839A > C	160,268,526
rs2481030	Intron variant −1296-7839A > C	160,268,526
rs316003	1506 A > G (V502V)	160,645,832
rs316024	Intron variant −1296-518G > A	160,261,205
rs316025	Intron variant −1296-1662A > T	160,262,349
rs316026	Intron variant −1296-2651G > A	160,263,338

Data were collected from the Ensembl database (https://www.ensembl.org)

Table 2 summarizes the SNPs within the *SLC22A2* gene that exhibit the highest deleterious potential, as assessed by the SIFT score, along with the SNPs predicted to have the most likely or possible damaging effects based on the PolyPhen score. These variants are of particular interest due to their potential impact on the function of *SLC22A2* and their implications for related biological processes.

**Table 2:** SNPs in the *SLC22A2* gene with the highest deleterious potential and the most likely or possible damaging effects

Variant ID	Alleles	GMAF allele	Global MAF	Source	AA	AA coord
rs199783132	G/A/T	A	< 0.001	dbSNP	R/C	186
rs199783132	G/A/T	A	< 0.001	dbSNP	R/S	186
rs199783132	G/A/T	A	< 0.001	dbSNP	R/C	207
rs199783132	G/A/T	A	< 0.001	dbSNP	R/S	207
rs556491698	G/A	A	< 0.001	dbSNP	S/L	313
rs556491698	G/A	A	< 0.001	dbSNP	S/L	334
rs184227446	G/A	A	< 0.001	dbSNP	T/M	357
rs8177516	G/A/T	A	0.003	dbSNP	R/S	400
rs568574662	G/A	A	< 0.001	dbSNP	R/C	403
rs369166212	G/A	A	< 0.001	dbSNP	P/L	496
rs45599131	A/C	C	< 0.001	dbSNP	L/W	330
rs45599131	A/C	C	< 0.001	dbSNP	L/W	351
rs576766802	C/G/T	T	< 0.001	dbSNP	V/L	115
rs576766802	C/G/T	T	< 0.001	dbSNP	V/L	136
rs57371881	C/T	T	< 0.001	dbSNP	R/H	155
rs57371881	C/T	T	< 0.001	dbSNP	R/H	176
rs576102206	A/T	T	< 0.001	dbSNP	F/Y	381
rs567153149	C/T	T	< 0.001	dbSNP	R/H	404

Data were collected from the Ensembl database (https://www.ensembl.org)

GMAF, Global minor allele frequency; AA coord, Amino acid location in the protein

In the *SLC22A2* coding region, several human genetic variants have been found and functionally defined [36]. In diverse ethnic groups in America and the Asian Pacific, researchers have discovered over 20 genetic polymorphisms and isoforms in the *SLC22A2* gene [43, 123]. While noncoding and promoter polymorphisms may have less effects, they can still affect the function of *SLC22A2*. Some of these variations in the coding region can lead to missense mutations, significantly affecting the role of the *SLC22A2* substrate transporter. Moreover, researchers examined several missense polymorphisms in individuals of Sub-Saharan African and non-African origins. Surprisingly, they found that three missense polymorphisms showed significant differences in frequency, while other polymorphisms had minor allele frequencies like those of other ethnic groups [18, 68]. Both rs138765638 and rs59695691 (promotor polymorphisms) considerably reduced the expression of the luciferase reporter gene compared to the wild-type control. These findings highlight the significance of locating and characterizing genetic variants in pharmacologically significant genes.

**Fig. 7:**
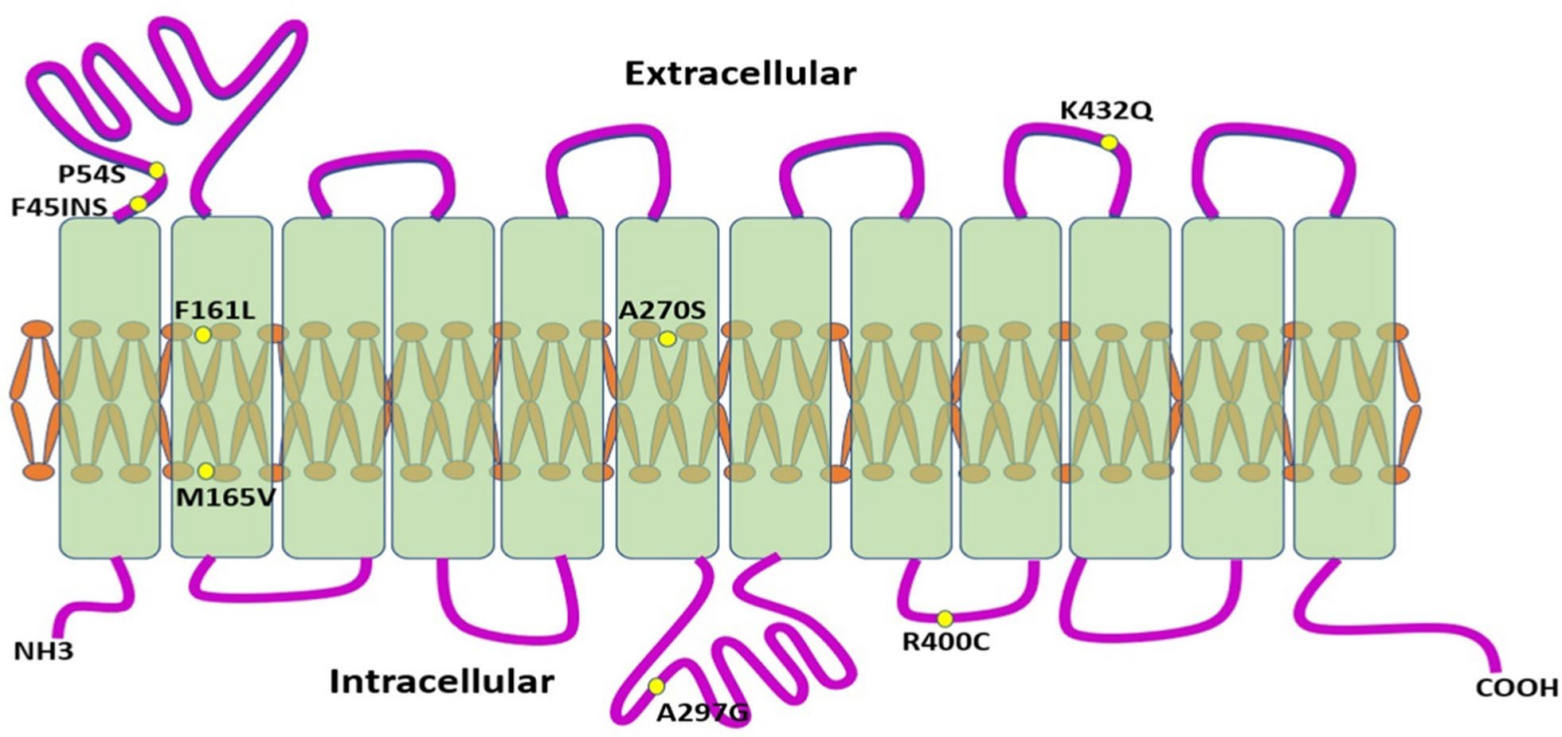
The most common and studied SNPs in the *SLC22A2* gene

Studies investigated many non-synonymous SNPs in the *SLC22A2* gene, including 495G > A (M165I), 808G > T (A270S), 1198 C > T (R400K), and 1294 A > C (K432Q) [123, 127]. The SNPs rs316019 (A270S), rs8177504 (P54S), rs8177509 (F161L) and rs8177508 (M165V), among others, are the most prevalent and studied [135], as shown in Fig. 7. Importantly, most pharmacogenetic studies have focused on SNP 808G > T (rs316019), the common coding polymorphism in *SLC22A2* that is present in more than 10% of all ethnicities [141]. The SNP rs316019 in exon 4 of the *SLC22A2* gene involves a 808G > T transversion that causes a serine-to-alanine substitution at codon 270, leading to a modest substrate-dependent reduction in OCT2 activity [43, 123, 127]. The *SLC22A2* variant 808G > T has been associated with reduced K_m_ and V_max_ values, as well as decreased absorption of its substrates, including dopamine [53, 138, 142]. Therefore, the relationship between this variant and metformin responsiveness in diabetic individuals has been extensively studied.

In one study, the non-synonymous rs316019 (808G > T) variant was associated with reduced tubular and renal disposition of metformin [123]. In this study involving 15 healthy Chinese adults, the 808G > T variant was shown to reduce creatinine clearance without significantly increasing overall drug exposure [123]. In another study, patients with the TT mutant genotype exhibited elevated concentrations of blood lactate compared to those in the non-metformin-treated group [67]. A Korean study also linked variations in the *SLC22A2* gene, including 596 C > T, 602 C > T and 808G > T, to impaired renal disposition and elevated plasma concentrations of metformin [110]. In contrast, a study on healthy volunteers of European and African ancestries demonstrated that 808G > T was associated with a greater renal clearance of metformin. Interestingly, the study observed that metformin levels were significantly higher in the reference group (808G/G) at earlier times after metformin administration. Therefore, lower plasma concentrations would be expected in individuals with the heterozygous for the variant allele (808G/T), who eliminate the drug more rapidly, in comparison to those who have the reference alleles (808G/G) [19]. These findings emphasize the importance of considering genetic variations in the *SLC22A2* gene across different ethnic groups when evaluating metformin metabolism and clinical response. However, further prospective studies are needed to fully understand the clinical significance of *SLC22A2* polymorphisms in relation to metformin efficacy.

### Structure and interactions of OCT2

Initial models of OCT structures were built using inward-facing templates oriented toward the cytoplasm. Subsequent outward-facing transporter models provided further insights into the complexity of ligand interactions with OCTs. Figure 6 presents a homology model of human OCT2. The structural prerequisites for drug interactions with OCT2 transporters are still not completely known, even though various drugs have been discovered to interact with human OCT2 [143]. OCTs are so-called because of the way that organic cations flow through the plasma membrane. The requirement for effective cation transport is the existence of a controlled anionic surface that attracts the cationic ligand on one side of the membrane, causes a conformational change in the transporter, and releases the substance on the opposite side of the membrane in a single efficient transport cycle. Several negatively charged residues (e.g., E232, D155, D382, E451, etc.) are found in OCT structures, some located near D22 and CORT molecules related to OCT3 [55].

The effect of *SLC22A2* 270 A > S mutation on interactions with metformin as well as other medications were studied computationally using molecular docking principles. The study discovered that the 270 A form of *SLC22A2* has a more decisive binding location for substrates and that the residue SER358 plays a crucial role in its interactions and affinity for metformin ligand pairing [103].

In a study investigating OCT1 and OCT2 orthologs from human, rat, mouse, and rabbit samples, multiple alignments revealed that most variations involved amino acids with similar mutation indices and antigenic site affinities [139]. However, some variations contained residues with distinct physicochemical properties. The study focused on the C-terminal half of OCT2, which is thought to influence the selectivity of OCT family members. The effect of mutations on OCT2 selectivity, specifically the residues N353L, R403I and E447Q, was examined in rabbit OCT2. While these mutations increased tetraethylammonium (TEA) substrate transport, there was only a slight and statistically insignificant increase in TEA affinity. The E447Q mutation was found to significantly impact OCT2 binding properties. IC_50_ values for the inhibition of TEA uptake by cimetidine and NBD-MTMA were unaffected by N353L and R403I alone, but E447Q caused a notable reduction in affinity, increasing the IC_50_ cimetidine by over 15 times. When both E447Q and N353I mutations were present, affinity for both inhibitors was further reduced. Structural analysis revealed that Glu447 is located in a hydrophilic cleft within the protein, influencing OCT2 binding [139]. In contrast, the E447L mutant preserved the ability to transport 1-methyl-4-phenylpyridinium but demonstrated dramatically lowered transport of cimetidine and TEA. There was a strong correlation between the calculated K_D_ values for 14 different compounds binding to the rabbit OCT2 model and their IC_50_ values for blocking TEA transport [139]. These findings highlight the critical role of specific mutations in modulating OCT2 binding and transport activity, which could have implications for understanding drug interactions and optimizing drug design.

### Potential effects of SLC22A2 variants in metformin-treated people

#### On renal elimination, disposition and therapeutic efficacy of metformin

Over the years, various tests, including fasting plasma glucose, glycated hemoglobin A1c (HbA1c), homeostasis model assessment of insulin sensitivity (HOMA-IS) and homeostasis model assessment of insulin resistance (HOMA-IR), have been used to evaluate the effectiveness of metformin [18]. Although the impact of genetic polymorphisms in *SLC22A1* and *SLC22A2* on metformin disposition has been established, they do not entirely account for the variations in clinical efficacy [107, 140]. Variations in the *SLC47A1, SLC47A2*, *SLC22A1,* and *SLC22A2* promoters were studied for their implications on metformin response in newly diagnosed T2DM patients in Chaoshan, China. While earlier studies had indicated the significant effects of *SLC47A1* rs2252281, *SLC22A2* rs316019, *SLC22A1* rs622342 and *SLC47A1* rs2289669 on metformin pharmacokinetics and pharmacodynamics [9, 22], the interrelationship between *SLC22A2* rs316019 and *SLC47A1* rs2289669 was not linked to insulin resistance in T2DM individuals receiving metformin treatment [18]. Additionally, a study on the genetic polymorphisms in *SLC22A2* discovered that *SLC22A2* variants, when compared with reference genotype, had significant differences in metformin pharmacokinetics, including higher peak plasma concentration, area under the curve and lower renal clearance. This shows that reduced renal clearance of metformin produced by the *SLC22A2* mutations resulted in elevated plasma concentrations and poor transport function [110, 117].

Specific variants, including 165M > I, 199 T > I, 201 T > M and 400R > C, reduced the metformin disposition activity when compared to the reference *SLC22A2*, meanwhile transporter function in vitro is less affected by the 270 A > S (808G > T) variant [141]. Most pharmacogenetic research in healthy individuals have concentrated on the latter since it is the typical coding mutation in *SLC22A2* with an allele frequency of roughly 15% across ethnic backgrounds. The results indicate that while homozygous TT genotype carriers of the *SLC22A2* 808G > T SNP exhibit a substantial 40% decrease in metformin disposition, heterozygous carriers of the *SLC22A2* 808G > T SNP do not experience a significant reduction in renal metformin clearance. These findings align with a recessive model of genotype–phenotype interaction, according to which 808 allele defects are required for changes in renal metformin clearance to occur. To obtain optimal glucose control, people having the *SLC22A2* 808 GG or GT genotype probably need to take a different dose of metformin than homozygous TT allele carriers [141].

A few carriers of the uncommon *SLC22A2* 596 C > T (199T > I) and 602 C > T (201T > M) polymorphisms, which demonstrate lowered metformin transport, were discovered through research in cellular models by Song et al. The metformin clearance of these carriers was compared to that of the wild-type allele, and it was discovered that renal metformin clearance was decreased to a level similar to that seen in homozygous carriers of the *SLC22A2* 808 TT variant when only one reduced-function *SLC22A2* 596 C > T or 602 C > T allele was present [110]. However, the small sample size in this study makes it challenging to reach firm conclusions. These SNPs only have a frequency of 1% in some ethnic subpopulations. Therefore, their clinical significance can be constrained when used in a population-based pharmacogenetic screening method.

In a different investigation, it was discovered that in transfected HEK-293 cells, *SLC22A2*−808T had a more remarkable metformin transport ability than the reference OCT2 [19]. The pharmacokinetics of metformin were assessed in 23 healthy volunteers constituting Caucasians and African Americans. It was found that healthy individuals homozygous for the 808 GG reference allele had significantly higher renal clearance and net secretion of metformin than volunteers carrying the heterozygous variant allele 808 GT [19]. In another study, 50 healthy volunteers with genotype 808G > T were recruited, with 5 TT, 25 GG and 20 GT individuals participating. The researchers examined the pharmacokinetics of a single oral metformin dose of 500 mg. The secretory clearance or renal clearance of metformin were unaffected by the 808G > T variation. Volunteers who had minor alleles in 808G > T and were homozygous for the reference variant 66 T > C (in *SLC47A1*), on the other hand, exhibited greater renal clearance and secretory clearance values than volunteers who had minor alleles in 808G > T but did not have the 66 T > C reference genotype [22]. The interactions between different SNPs and the renal clearance and secretory clearance of metformin, and efficacy is crucial in understanding the pharmacokinetics and individual response variability of metformin.

According to a Korean study, the *SLC22A2* 596 C > T, 602 C > T and 808G > T variants have been related to lower renal clearance and higher plasma levels of metformin [53]. Similarly, three intergenic variants between *SLC22A2* and *SLC22A3* (rs3119309, rs7757336 and rs2481030) were significantly linked to the ineffectiveness of metformin in a European cohort. Despite the noncoding nature of the discovered polymorphisms, they may be in linkage disequilibrium with causal SNPs in the coding or regulatory areas of *SLC22A1, SLC22A2* or *SLC22A3*, leading to a distorted transport activity or expression levels in the target tissues [134]. Nonetheless, a study on 103 healthy male Caucasian volunteers did not find any significant link between renal metformin disposition and several *SLC22A2* variants (rs10755577, rs17588242, Val502, rs315996, rs316019, rs17589858, Thr130, among others) [117].

Moreover, the pharmacokinetic profile of metformin can also be influenced by the formulation type. IR metformin produces higher peak plasma concentrations, potentially leading to transient transporter saturation and greater systemic accumulation in individuals with low-function *SLC22A2* variants. In contrast, XR formulations yield more gradual absorption, which may reduce transporter burden and mitigate some genotype-dependent differences in drug handling. These distinctions underscore the importance of considering both genetic and formulation factors when optimizing metformin therapy.

Indeed, studies investigating the influence of *SLC22A2* genetic polymorphism on metformin have yielded varied results. Some research found no significant connections, suggesting genes may not play a substantial role in the therapeutic effects of metformin. In contrast, other studies identified mechanisms leading to increased metformin clearance, resulting in lower concentrations in the bloodstream. Additionally, specific contexts were associated with decreased metformin clearance, indicating a complex interplay between genetics and medication response. These findings underscore the importance of personalized medication approaches tailored to individual genetic and physiological factors.

#### On insulin resistance

Since the increased likelihood of developing T2DM is linked to insulin resistance [114], it is also worth discussing the potential effect of *SLC22A2* on this condition. Insulin resistance, also referred to as impaired insulin sensitivity, occurs when liver, muscle, and adipose cells exhibit poor responsiveness to insulin and encounter difficulties in assimilating glucose from the bloodstream to produce energy [126]. However, little is known about the connection between insulin resistance and genetic variations of *SLC22A2*. Kashi et al. discovered that the *SLC22A2* 602 C > T variant, which has been demonstrated to affect the functionality of OCT2, impacted resistance to insulin and the function of beta cells [54]. According to the study, subjects with the minor T allele demonstrated considerably greater HOMA-IR scores compared to those who were homozygous for the C allele, indicating that these individuals had more insulin resistance. The study further revealed that subjects with the T allele demonstrated greater HOMA-BCF (homeostatic model assessment of beta cell function) levels, indicating that 602 C > T variant may have a role in changes in resistance to insulin and the activity of beta cells in type 2 diabetics using metformin [54].

While the association between *SLC22A2* genetic variations and insulin resistance remains an area of ongoing research, it is possible that these polymorphisms may significantly impact the effectiveness of metformin in managing T2DM. Understanding how OCT2 functionality influences both insulin sensitivity and beta cell function could lead to more personalized treatment approaches, improving outcomes for individuals with T2DM, especially those using metformin as a primary therapeutic agent.

#### On lactate levels

**Fig. 8:**
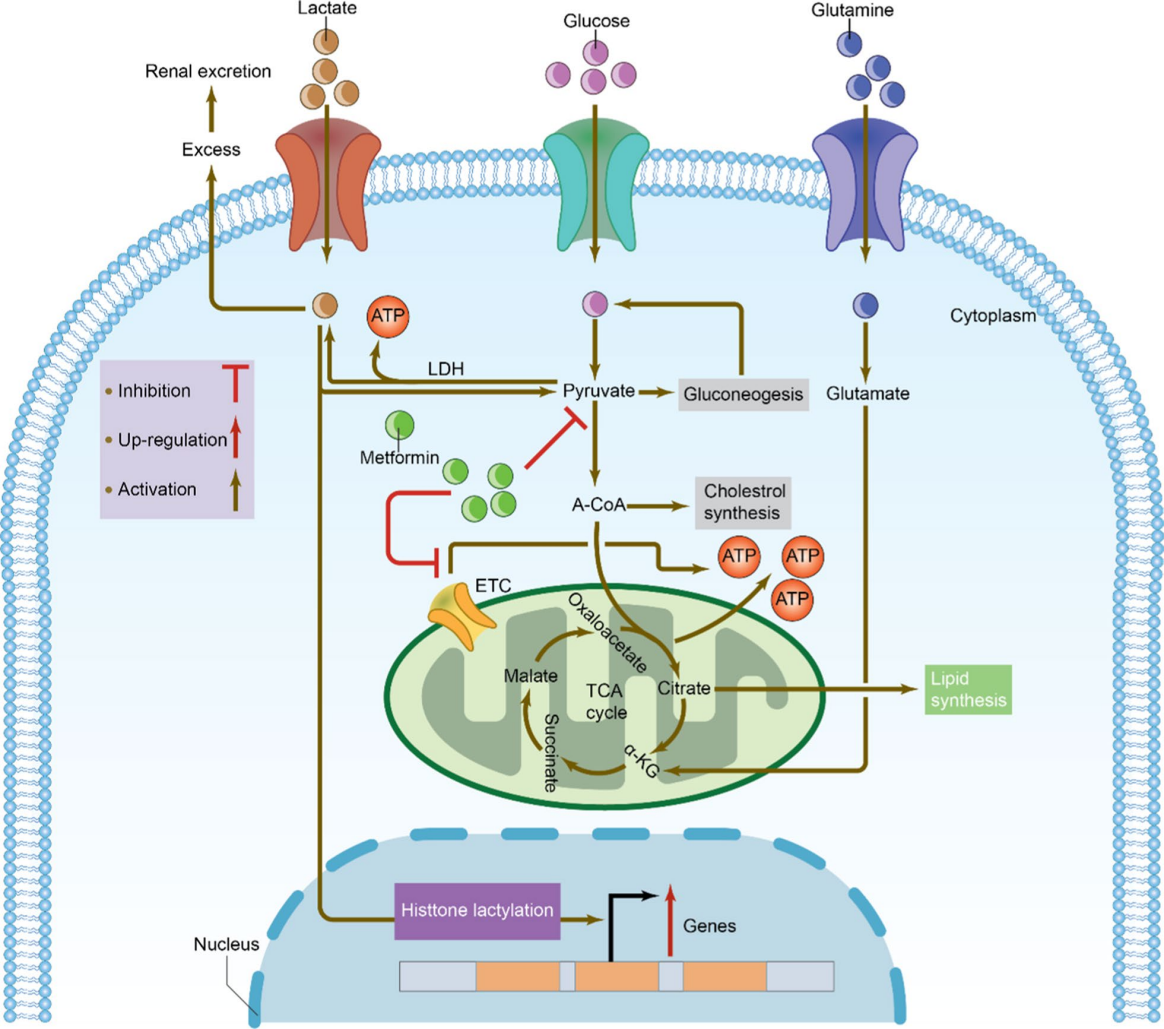
Mechanisms of metformin-associated lactic acidosis and lactate metabolism in cells. Metformin inhibits pyruvate carboxylase, impairing hepatic gluconeogenesis and leading to excess lactate. It also inhibits complex I of the mitochondrial electron transport chain (ETC), increasing the NADH/NAD + ratio and blocking pyruvate entry into the tricarboxylic acid (TCA) cycle. Nucleoside reverse transcriptase inhibitors (NRTIs) inhibit β-oxidation, the TCA cycle and DNA γ-polymerase, resulting in mitochondrial dysfunction and loss of transcription of essential enzymes, contributing to hepatic steatosis, myopathy, pancreatitis, nephrotoxicity, and lactic acidosis. Linezolid may disrupt mitochondrial protein synthesis involved in the ETC, cross-reacting with mammalian cellular processes. Propofol can inhibit coenzyme Q and cytochrome C at Complex IV in the ETC, as well as mitochondrial fatty acid metabolism. Isoniazid inhibits the metabolism of lactate to pyruvate. Lactate is transported into cells by monocarboxylate transporters (MCTs) and is produced from glycolysis or glutamine decomposition. It can be oxidized to pyruvate, which enters mitochondria and is metabolized through the TCA cycle, or converted to glucose via gluconeogenesis. Lactate is also converted into lactyl-CoA, participating in the lactylation of histones and nonhistone proteins. LDH, lactate dehydrogenase; A-CoA, acetyl-coenzyme A; α-KG, alpha-ketoglutarate

The equilibrium between lactate generation and clearance controls the amount of lactate in plasma. The liver uses lactate produced in several tissues like the gut, skin and red blood cells, to create glucose [12, 94]. Any excess lactate is eliminated through the kidney in the urine. There are two potential explanations for the metformin-induced rise in lactate levels: (1) a rise in lactate synthesis in peripheral tissues due to glycolysis and; (2) a reduction in lactate transport and processing in the hepatocyte and other organs [97]. Because complex I of the mitochondrial respiratory chain is inhibited by metformin, the flux through the Krebs cycle is decreased, and glycolysis is increased. The AMPK pathway increases the pyruvate level and partially inhibits gluconeogenesis, which causes more pyruvate to accumulate and more pyruvate to be converted to lactate. The G3P pathway is blocked by the inhibitory effect of metformin on mitochondrial glycerophosphate dehydrogenase (mGPD), which also affects the cytoplasmic redox state and prevents lactate from being converted to pyruvate, ultimately leading to metformin-associated lactic acidosis (MALA) [90]. As shown in Fig. 8, the mechanisms underlying MALA and the role of lactate metabolism in cellular processes are illustrated.

A small amount of ATP is generally produced by lactate metabolism (as shown in Fig. 8). The main organs metabolizing lactate are the hepatocytes and renal tissues, accounting for about 60 and 30% of lactate metabolic expulsion and clearance, respectively. Gluconeogenesis is the process by which lactate is turned back into glucose, or mitochondrial oxidation, in which lactate is transformed into carbon dioxide and water to provide energy. When lactate synthesis is excessive and hepatic lactate clearance is compromised, lactic acidosis can result, a potentially fatal medical condition defined by decreased arterial pH (7.35) and increased arterial levels of lactate (5.0 mEq/l in humans) [28, 122].

Two mechanisms have been identified for lactic acidosis: (1) increased lactate synthesis or decreased lactate clearance, such as in MALA and (2) lactate buildup via glycolysis in hypoxia. Increased plasma lactate levels caused by metformin are possibly caused by the inhibition of mitochondrial respiration in organs, such as the liver and muscles, responsible for lactate elimination [28]. Importantly, MALA is a complication with a mortality rate of 3–50% and could be fatal. An individual’s susceptibility to MALA may be increased by prior medical disorders such as renal dysfunction [103]. Therefore, T2DM patients who regularly take medications that lead to high levels of lactate accumulation in the blood should exercise caution.

Li and colleagues investigated the relationship between plasma lactate levels and the *SLC22A2* 808G > T (rs316019) genetic polymorphism in Chinese subjects with T2DM who were either treatment-naïve or were under metformin therapy [67]. They found significantly higher lactate levels in the treated group than in the treatment-naïve group. The OCT2 transporter could not bind to metformin and is released into the urine in patients with the T allele at position 808 of the *SLC22A2* gene, leading to a rise in metformin concentration and, subsequently, lactate generation. In the treated group, females with similar or identical *SLC22A2* 808G/T genotypes had greater plasma lactate concentrations than males. According to the study, compared to women with genotypes GG or GT, women with genotype TT had noticeably greater levels of plasma lactate [67]. These findings underscore the potential role of the *SLC22A2* polymorphisms in contributing to elevated plasma lactate levels, which may increase the risk of lactic acidosis in T2DM patients undergoing metformin therapy. However, studies investigating this association remain scarce, highlighting the need for further research to better understand the clinical implications.

### Drug interactions, metformin response and OCT2

As metformin is not metabolized in the liver, drug-drug interactions caused by the inhibition of different OCT transporters may be considerable and clinically relevant [87]. Therefore, it is recommended to evaluate pharmacokinetic interactions at both the preclinical and clinical stages, as these interactions could impact the safety and efficacy of combination therapies [46]. Understanding drug-drug interactions is crucial, especially with recent advancements in our knowledge of drug transporter functions in pharmacokinetics. Drug entry into or efflux from cells is mediated by uptake and efflux transporters. Since metformin is primarily transported via the OCTs in kidneys, drug interactions related to the inhibition of OCTs, especially OCT2, may be particularly significant for patients with T2DM taking metformin. Particularly, drug interactions can lower renal clearance and perhaps elevate the systemic distribution of metformin since OCT2 is present in the proximal tubule epithelium [107].

**Fig. 9:**
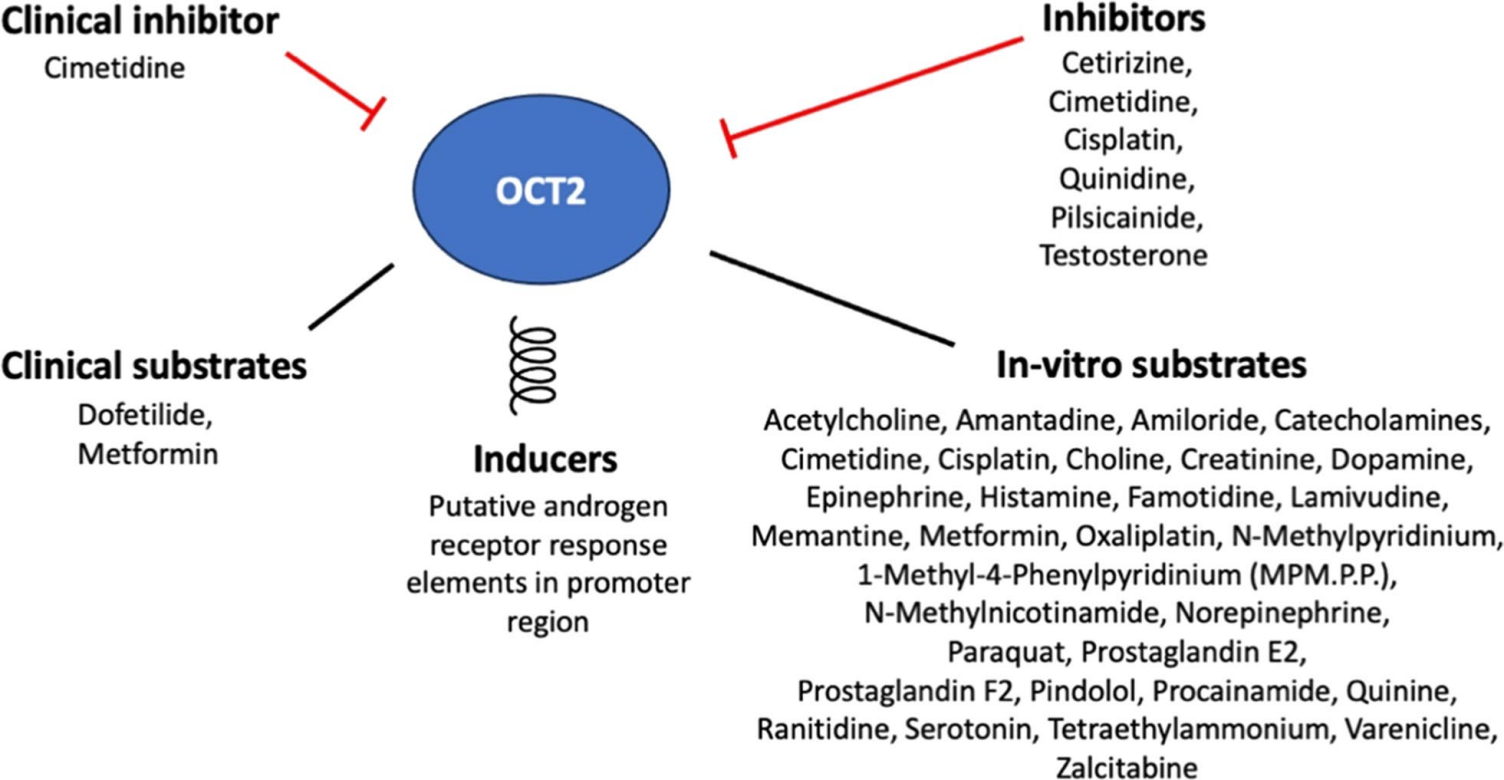
Overview of OCT2 substrates, inhibitors and inducers

For instance, the transport of common substrates, such as metformin, through renal tubular cells relies on the interaction between OCT2 and MATE1 [77]. The significant overlap in substrate selectivity between these transporters suggests they may share an affinity for pharmacological inhibitors, and their activities are likely coordinated [77]. Drugs like cimetidine, ranitidine and probenecid can inhibit both OCT2 and MATE1. These interactions may lead to elevated plasma levels of the substrate drug, increasing the risk of adverse effects, including lactic acidosis, in organs beyond the kidneys [77]. Figure 9 illustrates the key substrates, inhibitors, and potential inducers of OCT2, highlighting its critical role in drug transport and metabolism. Therefore, studying drug interactions is particularly crucial in understanding metformin response, especially with the rising prevalence of polypharmacy in patients with T2DM, who often take multiple medications for various comorbidities. Below, we discuss selected drugs and compounds that have the potential to interact with metformin.

### Cisplatin

Cisplatin, a commonly used chemotherapeutic agent, is actively transported into kidney cells via OCT2. It has the potential to induce renal dysfunction, which can impair metformin clearance by reducing its elimination through OCT2 [35, 89]. Given the role of OCT2 in modulating cisplatin metabolism and its effects, the factors that regulate its uptake and interaction with OCT2 remain unclear. While studies have investigated *SLC22A2* variants for potential mechanistic insights, alternative mechanisms that limit OCT2 expression may also be involved in cisplatin transport.

It was questionable whether genetic variations in *SLC22A2* significantly impacted the renal elimination of cisplatin, given the absence of a higher prevalence of rare variants in global populations and the apparent evolutionary selection against amino acid variants in OCT2 [65]. Useful information from heterologous expression models indicated that the *SLC22A2* 808G > T variant exhibits similar levels of expression and functional activity compared to the OCT2 reference protein [36]. Another study showed that the 808G > T variant does not appear to significantly affect the interindividual pharmacokinetic variability of cisplatin [33]. This suggests that only rare variants are likely to cause significant phenotypic changes, while other factors may contribute to modulating cisplatin uptake and its interaction with OCT2. These factors could potentially influence both cisplatin pharmacokinetics and metformin clearance.

On the other hand, it has been proposed that reducing cisplatin accumulation in the inner ear helps shield it from the drug adverse effects, thereby preventing cisplatin-induced ototoxicity. This is based on the assumption that OCT2 is expressed in the cochlea of humans, as it is in mice, and that the *SLC22A2* 808G > T variant has a decreased affinity for cisplatin [25, 80]. Then, another study found a strong correlation between the *SLC22A2* 808G > T variant and a reduced risk of cisplatin-induced ototoxicity in both pediatric and adult groups [64]. Although the researchers claimed that multiple logistic regression studies, which considered age as a significant predictor of ototoxicity, supported this conclusion, it remained unclear how individuals with the T allele experience less ototoxicity. Ultimately, it is still unknown how the *SLC22A2* 808G > T variant affects the expression of *SLC22A2* and OCT2 in human cochlea.

Understanding of the underlying genetic factors contributing to the pharmacokinetic and pharmacodynamic interactions between these two drugs can provide valuable insights into their combined effects and help optimize treatment strategies. By elucidating the underlying mechanisms and identifying specific genetic markers, personalized approaches to dosing and management can be developed, enhancing the efficacy and safety of metformin in individuals receiving cisplatin-based chemotherapy regimens.

### Cimetidine

Cimetidine has been demonstrated to reduce drug renal clearance by inhibiting renal OCT2-mediated drug transport [44]. A study by Müller et al. demonstrated that cimetidine and metformin interact via OCT2 and MATE1 in MDCK cells, which mimic renal drug transport. The study confirmed that OCT2 facilitates the uptake of cimetidine into renal cells, where it interacts with MATE1, influencing metformin transport and reducing its clearance [83]. A study on 112 healthy Chinese volunteers identified genetic variants in OCT2, including the 808G > T polymorphism. A pharmacokinetic experiment with 15 participants showed that cimetidine reduced metformin clearance, with the TT group experiencing a smaller reduction than the GG group. The findings established a link between the 808G > T polymorphism and reduced renal disposition of metformin, highlighting the mutation role in enabling cimetidine to inhibit the renal tubular transport of metformin [123].

While cimetidine was characterized as an OCT2 inhibitor drug by in vitro, in vivo and clinical studies [10], its inhibitory potential towards MATE1 was found to be approximately 225-fold and 170-fold more potent than its inhibition of OCT1 and OCT2, respectively [32]. A study by Yang et al. showed that the drug-drug interactions with six perpetrators including cimetidine, pyrimethamine, trimethoprim, ondansetron, rabeprazole and verapamil. The results revealed that inhibition of renal OCT2, renal MATEs, and gastrointestinal transit rate were the most critical factors influencing the drug interaction between cimetidine and metformin, while the most significant contributions to metformin disposition came from renal OCT2 and renal MATEs [133].

### Verapamil

Verapamil, a calcium channel blocker, is processed by cytochrome P450 enzymes in the liver [46]. It can inhibit OCT2 and has also been found to reduce fasting blood sugar levels. As a result, there is rising interest in drug combination with anti-diabetic medications like metformin to treat diabetes mellitus. Due to the ability of verapamil to inhibit OCT2, after either oral or intravenous administration of both medications to rats, there was reduction in renal disposition of metformin and a rise in systemic exposure (i.e., plasma concentration). In contrast, the pharmacokinetic profile of verapamil was unaffected by metformin [46].

### Naringenin

Naringenin is a flavonoid utilized traditionally in Chinese medicine for diabetes treatment, and it was studied for its effects on OCT1 and OCT2 protein expressions in the renal tubules of diabetic rats [131]. In diabetic rats, naringenin markedly reduced plasma metformin concentrations and boosted creatinine clearance compared to the control group. However, in rats administered naringenin or metformin alone, OCT1 and OCT2 protein expressions in renal tissue remained unaltered [131]. Naringenin and metformin combined administration elevated the expression of OCT2 in diabetic rats. Naringenin may improve renal metformin absorption and disposition by upregulating OCT2 protein expression, aiding metformin clearance in diabetic rats [75].

### Rifampin

A study aimed to find out how the pregnane X receptor (PXR) agonist, rifampin, affected the pharmacokinetics of metformin in 16 healthy volunteers. The mRNA levels of OCT1 and OCT2 were also measured using the oral glucose tolerance test (OGTT). After a 10-day course of rifampin, the OGTT was conducted on days 1 and 2, and on days 13 and 14. The area under the concentration–time curve and peak glucose levels significantly increased following glucose ingestion [20]. The systemic exposure of metformin was marginally elevated by 13%, while its renal clearance was elevated by 16%. However, rifampin boosted OCT1 mRNA levels but not OCT2 in peripheral blood cells. Further, rifampin was found to improved the glucose-lowering activity of metformin by boosting OCT1 expression in the liver [20].

### Moxifloxacin and ethambutol

Moxifloxacin and ethambutol are both antibiotics used to treat tuberculosis (TB). Although the likelihood of drug interactions between metformin and anti-TB drugs is not well understood, it is thought that OCTs and MATEs play a significant part in the metabolism of metformin. As a result, these transporters are likely to mediate any likely drug interactions [115]. In an in vitro study conducted to determine whether medications used to treat TB interferes with metformin transport and whether they serve as substrates for the MATE and OCT transporters, the effects of TB drugs on the absorption of metformin and the identification of transporter substrates were investigated using HEK-293 cells overexpressing MATEs and OCTs. The findings demonstrated that moxifloxacin was a strong inhibitor of MATE1- and MATE2K-mediated metformin transport. Only ethambutol was discovered to be a substrate of OCT1, OCT2, OCT3, MATE1, and MATE2K among all TB medications. These results offer a mechanistic foundation for anticipating ethambutol drug-drug interactions [115].

### Proton pump inhibitors

Although metformin is frequently combined with proton pump inhibitors (PPIs), it is unknown how much of an impact these interactions will have on OCTs [20]. In silico modeling and computational studies were used to create pharmacophore models to study this. The results showed that PPIs, such as omeprazole, rabeprazole, lansoprazole, pantoprazole, and tenatoprazole, demonstrate potent inhibition of OCTs [44, 87]. To test the effect of these PPIs on OCT-mediated metformin absorption, in vitro tests were performed utilizing cell lines stably transfected to express human uptake transporters (OCTs). The findings demonstrated that all PPIs tested significantly and concentration-dependently decreased metformin absorption by all the three OCTs, commensurate with those of other potent OCT inhibitors. However, PPIs were not recognized as OCT substrates. Additionally, pantoprazole was discovered as one of the best inhibitors of OCT2-mediated MPP + uptake [44, 87].

### Insights for other drugs

Perazine, aripiprazole, benperidol, rivastigmine, isosorbide dinitrate, among other frequently given pharmaceuticals, were among the substances with possible OCT2 inhibitory effects investigated in a study [44]. Using experimental probe substrates like ASP + (acetylsalicylic acid) or MPP + (1-methyl-4-phenylpyridinium), the inhibition was ascertained in vitro. Whether data on inhibition obtained with experimental probing substrates like ASP + or MPP + can be utilized to predict the inhibition against other substrates with therapeutic value, like metformin, has been questioned. The researchers, thus, compared the OCT2 inhibition characteristics of ASP +, MPP +, and metformin. The potential inhibitors of OCT2-mediated metformin and MPP + absorption was examined in human embryonic kidney cells that stably overexpress OCT2 [44]. The information on OCT2-mediated ASP + uptake inhibition was found in prior studies. It was discovered that there was a moderate correlation between the inhibition of OCT2-mediated MPP^+^, ASP^+^, and metformin uptake. The researchers identified OCT2 inhibitors that were both selective and universal and prevented the transport of more than 50% of either a particular substrate or all substrates. According to the study, employing experimental substrates instead of certain victim medications has less predictive value for drug-drug interactions. Pantoprazole, olanzapine, clomipramine, and doxepin were among the drugs studied [44]. At a concentration of 20 M, 45 out of the 125 medicines tested significantly reduced MPP + absorption. Thirteen medications showed excellent efficacy, IC_50_ values < 20 M, and more than 50% reduction in MPP + transport. The top four inhibitors of OCT2-mediated MPP + uptake were olanzapine, doxepin, clomipramine and pantoprazole [78]. According to its inhibitory profile, OCT2-mediated MPP + uptake was inhibited in a concentration-dependent manner. It changed towards more significant inhibition at a concentration of 200 M compared to 20 M. Contrarily, some of the studied medications, including hydrochlorothiazide, enhanced the absorption of metformin and MPP + through the OCT2-mediated pathway [44].

YM155 monobromide, a small-molecule inhibitor of survivin, was found in a study to exhibit a strong anticancer property in a prostate cancer mice model [78]. The substance enters hepatocytes and cancer cells through transporters because it has a cationic moiety in its composition. In this study, HEK-293 cells that had been engineered to express OCT1, OCT2 and OCT3 were used to investigate the transport of YM155. With IC_50_ values of 23.8, 15.9, and 108 µM, respectively, YM155 prevented the uptake of a typical substrate, 1-methyl-4-phenylpyridinium, through OCT1, OCT2 and OCT3 [109]. The absorption of YM155 was time- and concentration-dependent in OCT1- and OCT2-expressing cells, with K_m_ values of 22.1 µM and 2.67 µM, respectively. However, no significant absorption was observed in OCT3-expressing cells. The findings suggest that YM155 is taken up from the circulation into liver and proximal tubular cells in humans through OCT1 and OCT2, respectively, considering the tissue distribution and localization of each transporter [78].

## Future directions

The primary challenge in applying precision medicine to anti-diabetic treatment lies in identifying and understanding the diverse individualized factors that influence the response of patient to therapy. These factors encompass a wide range of variables, including genetic predispositions, epigenetic modifications, lifestyle habits, environmental exposures, microbiome composition and coexisting medical conditions [23, 38]. By unraveling the complex interplay of these elements, researchers and clinicians aim to develop personalized treatment strategies that optimize glycemic control, minimize complications, and improve both the quality and longevity of life for individuals with T2DM. Addressing this challenge requires an interdisciplinary approach, leveraging advancements in genomics, proteomics, metabolomics and big data analytics to tailor interventions to the unique profile of each patient.

Incorporating pharmacogenetic information offers a critical layer of patient stratification, enabling a more nuanced understanding of individual responses to medications [45]. Ongoing pharmacogenomics and metabolic studies are actively exploring the factors influencing individual responses to metformin, with insights from these investigations poised to inform future multi-omics research. Efforts should focus on building a comprehensive understanding of the genetic, metabolic and molecular markers that govern the efficacy and variability of metformin in different populations. This iterative process will refine the knowledge base required for precision medicine approaches, enabling the identification of specific subpopulations for deeper exploration. However, the adoption of pharmacogenomic guidance in clinical practice faces several challenges, including logistical hurdles (e.g., slow test turnaround times, timing issues at the point of care and limited access to certified laboratories) and financial barriers. Overcoming these challenges is critical to integrating pharmacogenomics into standard medical practice.

Future studies investigating *SLC22A2* variants, both previously studied and newly identified, should employ advanced techniques such as site-directed mutagenesis and computational modeling to unravel the mechanistic basis of how specific *SLC22A2* variants alter substrate affinity and transporter efficiency. Exploring the systemic effects of these variants on metabolic pathways will provide a more comprehensive understanding of their role in the response to metformin. Large-scale population studies are also essential to assess the prevalence of *SLC22A2* polymorphisms across diverse ethnic groups and their influence on the efficacy of metformin and other OCT2 substrates. These efforts will be crucial for refining personalized medicine strategies, enabling more precise drug dosing and improving treatment outcomes for patients carrying specific *SLC22A2* variants.

To strengthen the understanding of how *SLC22A2* polymorphisms, particularly 808G > T (A270S), influence insulin resistance outcomes beyond their established role in altering metformin pharmacokinetics, future research should focus on several key directions. Prospective longitudinal cohort studies that genotype patients for *SLC22A2* variants and monitor insulin sensitivity markers, such as HOMA-IR, fasting insulin, or euglycemic clamp data, over the course of metformin treatment are essential to establish temporal and clinical correlations. Functional studies using gene-edited cell lines or animal models carrying specific variants (e.g., A270S, R400C) would clarify the mechanistic effects of these mutations on metformin uptake, AMPK activation, and insulin signaling. Finally, gene–drug interaction trials stratified by *SLC22A2* genotype could assess differential IR outcomes under controlled metformin dosing regimens, with comparisons between immediate-release and extended-release formulations offering further clinical insight.

Moreover, the interactions of metformin with various drugs and substances, which influence its efficacy and safety profile, represent another crucial consideration. Understanding these interactions is important for advancing precision diabetes medicine, as it highlights the need for personalized treatment strategies that account for the complete medication profile of patients, genetic background and lifestyle factors. Future research should focus on utilizing multi-omics approaches to systematically predict and manage these interactions. By integrating these datasets with advanced bioinformatics tools and machine learning algorithms, researchers can identify biomarkers that modulate the pharmacodynamics and pharmacokinetics of metformin. Also, conducting longitudinal studies and clinical trials to assess the impact of drug-drug interactions in diverse populations could provide actionable insights to refine dosing guidelines.

## Conclusion

The genetic variability of OCT2 gene *SLC22A2* can play a crucial role in determining the pharmacokinetics and pharmacodynamics of metformin, influencing both its therapeutic efficacy and the risk of adverse effects in individuals with T2DM. The interindividual differences in these transporters, as well as drug-target proteins, contribute to significant variability in metformin response, particularly in patients with multiple comorbidities and polypharmacy. Therefore, incorporating genetic information into clinical practice is essential for optimizing metformin therapy. Further research is needed to fully understand the underlying mechanisms of these genetic interactions and to advance personalized medicine approaches that improve the efficacy of metformin treatment in T2DM patients. 

## Data Availability

Not applicable.
